# The complete mitochondrial genome of *Salmacis sphaeroides variegate* (Mortensen, 1942)

**DOI:** 10.1080/23802359.2019.1684215

**Published:** 2019-11-05

**Authors:** Wenfei Zhao, Zhe Li, Xiaofang Huang, Yang Zhang, Jun Ding, Yaqing Chang

**Affiliations:** Key Laboratory of Mariculture & Stock Enhancement in North China’s Sea, Ministry of Agriculture and Rural Affrairs, Dalian Ocean University, Dalian, Liaoning, P. R. China

**Keywords:** Mitochondrial genome, sea urchins, *Salmacis sphaeroides variegate*

## Abstract

In this study, the complete mitochondrial genome of *Salmacis sphaeroides variegate* was determined on Illumina HiSeq platform. The genome was 15,770 bp in size and contains 22 tRNA genes, 13 protein-coding genes, 2 rRNA genes, and 1 control region (180 bp). The composition of A + T in *S. sphaeroides* mtDNA was 61.90%. Except *ND6* and 6 tRNAs, the others are on the H-strand. The phylogenetic relationships of 11 species of sea urchins were analyzed using the neighbor-joining method using software MEGA 7.0. *S. sphaeroides* was most closely related to *Temnopleurus hardwickii*.

*Salmacis sphaeroides variegate* is a kind of sea urchin in the family Temnopleuridae. It lives in warm temperate regions and shallow waters (0–90 m). Although its nutritional value is very high (Chen et al. 2010), *S. sphaeroides* is not cultivated on a large scale in China. At present, the research on *S. sphaeroides* mainly focuses on biological development (Rahman et al. 2012, 2016). By comparing with the mitochondrial genome of other sea urchins, it provides basic data and important scientific data for the phylogenetic evolution and diversity of sea urchins. Therefore, the research on the mitochondrial whole genome of *S. sphaeroides* becomes very important.

Our sample was collected from Sanya, Hainan province, China (20°06′08.3″N,110°16′15.0″E). The complete mitochondrial genome of *S. sphaeroides* was determined using on Illumina HiSeq platform. Using SOAP denovo V2.04 software (http://soap.genomics.org.cn/), the sequence was assembled (Luo et al. 2012). DOGMA software (http://dogma.ccbb.utexas.edu/) was used to predict gene, rRNA, and tRNA contained in the genome. MEG A7.0 (Kumar et al. 2016) was used for multiple alignments. The mtDNA map of *S. sphaeroides* mitochondrial genome was drawn with the online tool OGDraw (https://chlorobox.mpimp-golm.mpg.de/OGDraw.html) (Lohse et al. 2013). The specimen is stored in the Key Laboratory of Mariculture & Stock Enhancement in North China’s Sea, Ministry of Agriculture and Rural Affrairs, Dalian Ocean University (voucher number: DLOU-KLM-SU05).

The mitochondrial genome of *S. sphaeroides* has a total length of 15,770 bp (GenBank registration number: MK692949), including 13 protein-coding genes, 22 tRNA genes, 2 rRNA genes, and a non-coding region with a length of 180 bp, which is consistent with the mitochondrial genome structure of the echinacea reported (Cantatore et al. 1989; Giorgi et al. 1996). The composition of A + T in *S. sphaeroides* mtDNA was 61.90%. Except *ND6* and 6 tRNAs, the others are on the H-strand. Most protein-coding genes (10 of 13 genes) started with ATG, *ATP8* with GTG, and *ND4* with ATC. The termination codon of most protein-coding genes was TAG (8 of 13 genes) or TAA (3 of 13 genes). The termination codon of *ND4* was TAT and the termination codon of *ND1* was TTA. The size of 22 tRNA coding genes ranged from 68 to 74 bp. The largest intergenic region of the mitochondrial genome was 36 bp.

The phylogenetic relationship of 11 species of sea urchins was analyzed using the neighbor-joining method by MEGA 7.0. The results showed that the *S. sphaeroides* were most closely related to the *Temnopleurus hardwickii*. The distance between Strongylocentrotus sea urchin and *S. sphaeroides* was the farthest (Figure 1).

**Figure 1.**
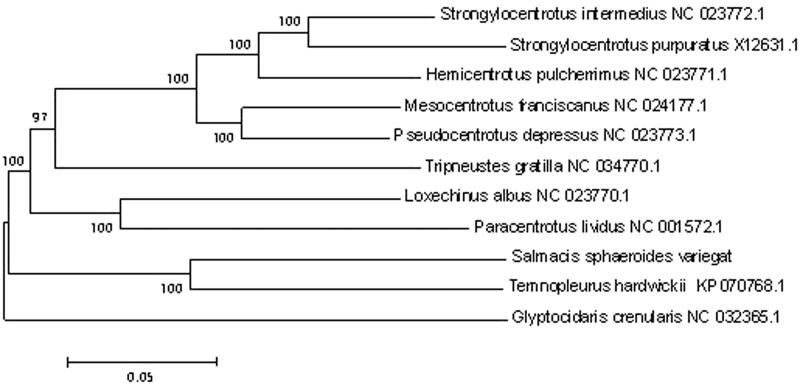
Consensus neighbor-joining tree based on the complete mitochondrial sequence of *S. sphaeroides* and other 10 species of sea urchins. The phylogenetic tree was constructed using MEGA 5.0 software by the neighbor-joining method. The numbers at the tree nodes indicate the percentage of bootstrapping after 1000 replicates.

The authors report no conflicts of interest. The authors alone are responsible for the content and writing of the article.

## References

[CIT0001] Cantatore P, Roberti M, Rainaldi G, Gadaleta MN, Saccone C 1989 The complete nucleotide sequence, gene organization, and genetic code of the mitochondrial genome of *Paracentrotus lividus*. J Biol Chem. 264(19):10965–10975. 2544576

[CIT0002] Chen G, Xiang W-Z, Lau C-C, Peng J, Qiu J-W, Chen F, Jiang Y 2010 A comparative analysis of lipid and carotenoid composition of the gonads of anthocidaris crassispina, diadema setosum and salmacis sphaeroides. Food Chem. 120(4):973–977.

[CIT0003] Giorgi CD, Martiradonna A, Lanave C, Saccone C 1996 Complete sequence of the mitochondrial dna in the sea urchinarbacia lixula:conserved features of the echinoid mitochondrial genome. Mol Phylogenet Evol. 5(2):323–332. 8728390 10.1006/mpev.1996.0027

[CIT0004] Kumar S, Stecher G, Tamura K 2016 Mega7: molecular evolutionary genetics analysis version 7.0 for bigger datasets. Mol Biol Evol. (7):1870–1874. 27004904 10.1093/molbev/msw054 PMC8210823

[CIT0005] Lohse M, Drechsel O, Kahlau S, Bock R 2013 Organellargenomedraw–a suite of tools for generating physical maps of plastid and mitochondrial genomes and visualizing expression data sets. Nucl Acids Res. 41(W1):W575–W581. 23609545 10.1093/nar/gkt289 PMC3692101

[CIT0006] Luo R, Liu B, Xie Y, Li Z, Huang W, Yuan J, He G, Chen Y, Pan Q, Liu Y, et al. 2012 Soapdenovo2: an empirically improved memory-efficient short-read de novo assembler. GigaSci. 1(1):18. 10.1186/2047-217X-1-18 PMC3626529 23587118

[CIT0007] Rahman MA, Yusoff FM, Arshad A, Ara R 2016 Growth and survival of the tropical sea urchin, salmacis sphaeroides fed with different macroalgae in captive rearing condition. J Env Biol. 37(4 Spec No):855. 28779748

[CIT0008] Rahman MA, Yusoff FM, Arshad A, Shamsudin MN, Amin SM 2012 Embryonic, larval, and early juvenile development of the tropical sea urchin, salmacis sphaeroides (echinodermata: echinoidea). Sci World J. 2012(4):938482. 10.1100/2012/938482 PMC3465900 23055824

